# Neonatal outcomes among multiple births ≤ 32 weeks gestational age: Does mode of conception have an impact? A Cohort Study

**DOI:** 10.1186/1471-2431-11-54

**Published:** 2011-06-14

**Authors:** Vibhuti Shah, Haydi AlWassia, Karan Shah, Woojin Yoon, Prakeshkumar Shah

**Affiliations:** 1 Department of Paediatrics, Room 775A, Mount Sinai Hospital, 600 University Avenue, Toronto, Ontario, M5G 1X5, Canada; 2 Woojin Yoon, MiCare Research Centre, 700 University Avenue, Room 8-500, Toronto, Ontario, M5G 1X6, Canada

**Keywords:** Assisted reproductive technology, in-vitro fertilization, spontaneous conception, infant-newborn, mortality, morbidity

## Abstract

**Background:**

Studies comparing perinatal outcomes in multiples conceived following the use of artificial reproductive technologies (ART) vs. spontaneous conception (SC) have reported conflicting results in terms of mortality and morbidity. Therefore, the objective of our study was to compare composite outcome of mortality and severe neonatal morbidities amongst preterm multiple births ≤ 32 weeks gestation infant born following ART vs. SC.

**Methods:**

We conducted a single center cohort study at Mount Sinai Hospital, Toronto, Ontario, Canada. Data on all preterm multiple births (≤ 32 weeks GA) discharged between July 2005 and June 2008 were retrospectively collected from a prospective database at our centre. Details regarding mode of conception were collected retrospectively from maternal health records. Preterm multiple births were categorized into those born following ART vs. SC. Composite outcome was defined as combination of death or any of the three neonatal morbidities (grade 3/4 intraventricular hemorrhage or periventricular leukomalacia; retinopathy of prematurity > stage 2 or chronic lung disease). Univariate and multivariate regression analysis were preformed after adjustment of confounders (maternal age, parity, triplets, gestational age, sex, and small for gestational age).

**Results:**

One hundred and thirty seven neonates were born following use of ART and 233 following SC. The unadjusted composite outcome rate was significantly higher in preterm multiples born following ART vs. SC [43.1% vs. 26.6%, p = 0.001; OR 1.98 (95% CI 1.13, 3.45)]; however, when adjusted for confounders the difference between groups was not statistically significant [OR 1.39, 95% CI 0.67, 2.89].

**Conclusion:**

In our population of preterm multiple births, the mode of conception had no detectable effect on the adjusted composite neonatal outcome of mortality and/or three neonatal morbidities.

## Background

Advances in neonatal intensive care have lead to increased survival of preterm [< 37 weeks' gestational age (GA)] infants at extremes of GA with its associated morbidities [1,2]. Over the last two decades there has been a steady increase in the incidence of preterm births worldwide with a range of 7-13% [3,4]. One of the reasons attributed to this increase has been the widespread use of assisted reproductive techniques (ART) [5,6] leading to pregnancies with multiple gestations (e.g. twins and triplets). In Canada in 2009, 29.1% of twin pregnancies and 0.9% of triplets and higher order pregnancies occurred following the use of ART [6]. Similarly for the United States, ART accounted for > 1% of all births and 18% of multiple births [7,8]. Studies comparing perinatal outcomes in multiples conceived following ART vs. spontaneous conception (SC) have reported conflicting results in terms of mortality and morbidity [9-11]. Factors that might have contributed to these conflicting findings include: inclusion of term and preterm infants in the cohort, studies with small sample size and failure to control for perinatal characteristics [12,13].

To date, there have been two published reports comparing outcomes of very low birth weight (VLBW) infants (≤ 1500 grams) conceived following ART vs. SC. Schimmel et al [14] reported that the risk of congenital malformations, mortality and neonatal morbidities [respiratory distress syndrome (RDS), patent ductus arteriosus (PDA), necrotizing enterocolitis (NEC), intraventricular hemorrhage (grades III/IV) and bronchopulmonary dysplasia (BPD)] were not increased in infants conceived by in vitro fertilization (IVF). Similarly, Messerschmidt et al [15] reported no difference in mortality, short-term pulmonary morbidity defined as the presence or absence of respiratory distress requiring the use of mechanical ventilation and surfactant administration and cerebral morbidity [grades 3/4 IVH or cystic periventricular leucomalacia (PVL)] among VLBW infants after IVF conception. There were no differences identified even when the group was divided in BW < 1000 g and 1000-1499 g. In both of these studies, outcomes were compared amongst singletons and multiples (twins and triplets) following ART vs. SC conception. However, increased neonatal morbidity has been attributed to plurality [6] and hence in our study we focused on mortality and severe neonatal morbidities amongst multiple births ≤ 32 weeks gestation infants born following ART vs. SC.

The objective of this study was to compare the composite outcome (death or severe neonatal morbidities) among multiple births ≤ 32 weeks GA born following ART vs. SC. We hypothesized that preterm multiple births ≤ 32 weeks GA born following the use of ART will have increased rate of adverse composite outcome. For the purpose of this study ART was defined as the use of artificial (infertility treatments in which both the oocytes and sperms are manipulated) or partially artificial (intracytoplasmic sperm injection and intrauterine insemination) methods to achieve pregnancy.

## Methods

We conducted a single center cohort study at Mount Sinai Hospital, Toronto, Ontario, Canada. Mount Sinai Hospital is a high-risk perinatal center and infants included in the study were inborn. Once the infants are stable (i.e. off ventilatory support) they may be transferred to the community hospital closer to home. Data were retrospectively collected from a prospective database for all preterm multiple births discharged between July 2005 and June 2008. Data on all NICU admissions were collected daily by either staff neonatologist or trained data abstractor according to predefined definitions and guidelines. Information on daily progress and clinical status, need for intensive care, resource utilization, cranial ultrasound scan and eye examination were collected. Data regarding maternal demographics, type of ART, use of antenatal steroids, maternal hypertension and diabetes during pregnancy and mode of delivery were collected from maternal health records.

### Study population

Preterm multiple births born at ≤ 32 weeks GA were included in this study. Neonates were divided into two groups: those conceived following the use of ART and those conceived spontaneously. Assisted reproductive technology was defined as the use of artificial (infertility treatments in which both the oocytes and sperms are manipulated) or partially artificial (intracytoplasmic sperm injection and intrauterine insemination) methods to achieve pregnancy. Infants with major congenital anomalies were excluded as it could influence our primary outcome [16]. The local research ethics board at our institution approved the database and this study.

### Outcomes

The primary outcome of our study was the composite outcome of mortality or any of the three neonatal morbidities [grade 3/4 IVH, or PVL, retinopathy of prematurity (ROP) > stage 2 or chronic lung disease (CLD)]. Secondary outcomes included individual components of the primary outcome, duration of positive pressure ventilation, duration of hospital stay, incidence of PDA, sepsis and NEC. Gestational age was defined as the best obstetric estimate based on early prenatal ultrasound, obstetric examination, and obstetric history. An infant was defined as small-for-gestational age (SGA) if the birth weight was less than the 3^rd ^percentile for GA according to the published data for Canadian infants [17]. Transport risk index of physiologic stability (TRIPS) scores were calculated from variables during the first 12 hours of admission to the NICU [18]. Mortality was defined as death occurring before discharge from the NICU. Information on death is not ascertained once the infant is transferred or discharged from our unit due to confidentiality issues. Further, the timing of death in both groups was delineated. Chronic lung disease was defined as oxygen dependency at 36 weeks corrected GA (CGA). Infants who were discharged from the NICU on oxygen before they reached 36 weeks CGA were classified as having CLD. Infants who died before they reached 36 weeks CGA were excluded from the analysis for CLD. Intraventricular hemorrhage was diagnosed according to the criteria of Papile from the worst findings on head ultrasound for all infants who underwent a head ultrasound after 24 hours of life [19]. Necrotizing enterocolitis was defined according to Bell's criteria (stage 2 or higher) [20]. Sepsis was defined as isolation of organisms from a normally sterile site (blood, urine or cerebrospinal fluid). Patent ductus arteriosus was diagnosed clinically based on symptoms and signs. The outcomes of NEC, sepsis and PDA were ascertained at any time during their hospital stay. Retinopathy of prematurity (ROP) was classified according to international classification [21]. Infants were eligible to undergo screening for ROP if they were born at ≤ 30 weeks GA or with birth weight ≤ 1,500 grams. They would be screened starting from 4-6 weeks postnatal age as per the Canadian Paediatric Society recommendations [22,23].

### Statistical analysis

Demographic characteristics and neonatal outcomes between groups were compared using χ^2 ^or Fisher's exact test for categorical data and Student's t-test or Mann-Whitney test for continuous data as appropriate. Odds ratio (OR) and 95% confidence interval (CI) was reported as appropriate. Logistic regression analysis was used to assess the independent effect of ART on composite outcome (death and/or any of the three neonatal morbidities) after adjusting for the following confounding variables: maternal age, parity, multiple type (triplets), GA, sex and SGA status. These variables were selected as they were noted to be statistically significantly different between groups on univariate analysis. However, as deaths in the delivery room were included in our analysis, infants who died in the delivery room were most likely to have low Apgar score at 5 minutes. Therefore, it may be an intervening variable in the model. Hence, logistic regression analysis was performed by including and excluding Apgar score at 5 minutes in the model. As there was no difference in the results in the two models, we present the results from the model that excluded Apgar score at 5 minutes. Generalized estimating equations (GEE) were used to control for correlation between multiples born to the same mothers. Statistical analysis was performed using SAS version 9.2 (SAS institute, Cary, NC). A p-value of < 0.05 was considered significant.

## Results

A total of 3208 infants were admitted to our NICU during the study period. Eleven hundred and thirty infants were ≤ 32 weeks gestation at the time of birth of which 370 were preterm multiples. One hundred and thirty-seven infants (from 65 mothers) were born following conception through ART and 233 (from 128 mothers) were born following SC. Baseline maternal and neonatal characteristics are presented in Tables 1 and 2 respectively. Maternal age was significantly higher [33.5 (5.3) vs. 29.5 (5.8) years; p < 0.01], mothers were more likely to be primiparous, and the rate of triplets was higher in the ART group (p < 0.01). The mean GA and birth weight were significantly lower in multiples born following ART vs. SC [28 (2.6) vs. 29 (2.2) weeks; p < 0.01] and [1099 (391) vs. 1213 (375) grams; p < 0.01] respectively. The distributions of the Apgar scores at 5 minutes were significantly different between ART vs. SC group (p < 0.01). The type of ART used are as follows: IVF (81%), intracytoplasmic sperm injection (4%) and intrauterine insemination (15%).

**Table 1:** Baseline maternal characteristics

Variable*	Assisted reproductive technology (ART) group (N = 65)	Spontaneous conception (SC) group (N = 128)	p value
Maternal age (years)	33.4 (5.4)	29.3 (5.7)	< 0.01

Gravida [median (range)]	1 (1, 9)	2 (1, 8)	0.01

Parity [median (range)]	0 (0, 3)	0 (0, 6)	< 0.01

Gestational diabetes [N (%)]	6 (9.2%)	6 (4.7%)	0.22

Hypertension [N (%)]	8 (12.3%)	15 (11.7%)	0.90

Use of antenatal steroid [N (%)]	59 (92.2%)	111 (87.4%)	0.31

Multiple type			
Twins	46 (70.8%)	119 (93%)	< 0.001
Triplets	18 (28.1%)	9 (7%)	
Quadruplets	1 (1.5%)	0	

*Results are presented as mean (SD) or median (range) or number [N (%)] as appropriate

**Table 2:** Baseline neonatal characteristics

Variable*	Preterm multiple births following ART (N = 137)	Preterm multiple births following SC (N = 233)	p value
Gestational age (weeks)	28 (2.6)	29 (2.2)	< 0.01

Birth weight (grams)	1099 (391)	1213 (375)	< 0.01

Male [N (%)]	76 (55.5%)	148 (63.5%)	0.12

Small for gestational age [N (%)]	8 (5.8%)	25 (10.7%)	0.11

Caesarean section (%)	81 (59.1%)	138 (59.2%)	0.98

Apgar score (1 minute)	5 (0, 9)	7 (0, 9)	< 0.01

Apgar score (5 minutes)	8 (1, 10)	8 (1, 10)	< 0.01

TRIPS at birth	14 (0, 55)	12 (0, 65)	0.01

TRIPS at 12 hours of age	14 (0, 31)	14 (0, 44)	0.28

*Results are presented as mean (SD) or median (range) or number [N (%)] as appropriate

TRIPS, transport risk index of physiologic stability

The composite outcome rate was significantly higher in the ART group [(43.1% vs. 26.6%, p < 0.01; unadjusted OR 1.98, 95% CI 1.13, 3.45]. When adjusted for confounding factors there was no significant difference in the composite outcome rate between the 2 groups [OR 1.39, 95% CI 0.67, 2.89]. Significant predictors for the composite outcome were: GA (p < 0.01) and being SGA (p < 0.03). Maternal age, parity, multiple type (triplets) and sex did not influence the composite outcome. Mortality and incidence of CLD were significantly higher in the ART group (p < 0.05). The incidences of PDA, NEC, sepsis, IVH and ROP were similar in both groups (Table 3). There were no differences in the duration of ventilation and length of hospital stay between the 2 groups (p > 0. 05).

**Table 3:** Neonatal outcomes

Variables*	Preterm multiples born following ART (N = 137)	Multiples born following SC (N = 233)	p value
**Composite outcome**†			

Death or any of the three severe neonatal morbidities [N (%)]	59/137 (43.1%)	62/233 (26.6%)	< 0.01

**Individual outcomes on mortality and neonatal morbidities**			

Mortality [N (%)]	28/137 (20.4%)	27/233 (11.6%)	0.02

Timing of infant death			

Within 1 day	12 (42.9%)	7 (25.9%)	0.49

2 - 6 days	6 (21.4%)	10 (37.0%)	

7 - 27 days	9 (32.1%)	8 (29.6%)	

≥ 28 days	1 (3.6%)	2 (7.4%)	

CLD^a ^[N (%)]	32/110 (29.1%)	31/206 (15.1%)	0.01

ROP > stage 2^b ^[N (%)]	9/90 (10.0%)	8/155 (5.2%)	0.15

Grade 3/4 IVH/PVL^c ^[N (%)]	12/125 (9.6%)	14/226 (6.2%)	0.24

PDA [N (%)]	35/137 (25.6%)	41/233 (17.6%)	0.06

NEC [N (%)]	7/137 (5.1%)	9/233 (4%)	0.58

Sepsis [N (%)]	25/137 (18.3%)	36/233 (15.5%)	0.48

Duration of positive pressure ventilation (days)	6 (0, 101)	9 (0, 149)	0.08

Length of stay (days)	24 (1, 144)	25 (1, 165)	0.10

*Results are presented as number [N (%)] or median (range) as appropriate

†Composite outcome includes mortality or any of the three neonatal morbidities (grade 3/4 IVH or PVL, retinopathy of prematurity (ROP) > stage 2 or CLD)

Denominator for each variable is as follows

^a^Infants on whom the outcome were ascertained (explanation in the methods section)

^b^Infants who were eligible for screening

^c^Infants who are alive for more than 24 hours

CLD, chronic lung disease; ROP, retinopathy of prematurity; PVL, periventricular leucomalacia, PDA, patent ductus arteriosus; NEC, necrotizing enterocolitis

## Discussion

In our study, the mode of conception did not influence neonatal mortality and morbidities in preterm multiples born at ≤ 32 weeks GA. Our findings are consistent with the studies published by Schimmel et a [14] and Messer Schmidt et al [15] who compared outcomes of singleton and multiples VLBW infants conceived by IVF compared to SC. After accounting for confounding variables (maternal age, parity, multiple type, GA, sex, and SGA) there was no difference in the incidence of neonatal morbidity or mortality among IVF-conceived infants. Regression analysis in our study revealed that GA and being SGA contributed to the adverse neonatal outcome. Further, infants in the ART group had higher mortality and incidence of CLD which can be explained by a one week difference in the GA between the two groups.

Maternal characteristics were different between the two groups for age, parity and type of multiples. Women in the ART group were more likely to be older and primiparous at the time of conception. This finding is consistent with other published studies in the literature [11,14,24,25]. There was no statistically significant increase in the use of antenatal steroids in women who conceived after ART vs. SC. In a case-control study on twins, Nassar et al [26] reported higher use of antenatal steroid in the IVF group compared to SC group, however that did not achieve statistical significance. Our rates of Caesarean section were not different between groups which are in contrast to what is reported in the published literature [12,26]. Variation in obstetrical practices amongst centers may account for the differences observed in our study. It is likely that women who conceive through ART receive close follow-up and are managed more aggressively [11].

Since the birth of the first IVF conceived infant in 1978, diverse ART techniques have developed rapidly and implemented in clinical practice. However, despite its adoption in practice no systematic effort has been made to scrutinize the safety of such treatments in relation to perinatal outcomes. To date, however, several systematic reviews and meta-analysis have been published on this topic [27-29]. The risk of preterm birth, LBW and VLBW were significantly increased in singleton pregnancies conceived by ART [27,28]. However, these findings should be interpreted with caution. Limitations of the published studies include: a) clinicians were not blinded to the mode of conception resulting in differential management of ART pregnancy with a low threshold to intervene if obstetrical complications arose; b) the pregnancy may have begun as a multi-fetal pregnancy with fetal loss in the first trimester which is associated with adverse perinatal outcomes and c) not all studies accounted for confounders. Similarly, McDonald et al [29] reported an increased risk of preterm birth for twin pregnancies in the IVF group. However, this finding was not replicated in a population based study when adjusted for socioeconomic status [11]. The comparable outcome may be attributed to enhanced monitoring and perinatal care in ART pregnancies.

The precise mechanism by which ART may increase the risk of preterm birth is not clear. Suggested mechanisms include: the infertility treatment, the cause of infertility (reproductive pathology in the couple themselves) or may be related to physician or patient anxiety (iatrogenic early delivery). Increased levels of relaxin, a powerful stimulator of collagen breakdown has been observed in gonadotropin stimulated pregnancies which persists throughout the pregnancy. It has been identified as an independent risk factor for preterm birth as it leads to premature cervical changes and delivery [30].

There are several limitations to our study. It is possible that women in the SC group may have received treatment such as the use of ovulation inducing agents which could influence our results towards the null hypothesis (i.e. no difference in the outcome). We were unable to evaluate the influence of chorionicity on outcomes between the groups as this information was not consistently recorded in the maternal charts. In this study we excluded singletons; therefore we are unable to comment on the influence of plurality on the composite outcome. Further, the limited sample size in our study may account for our inability to demonstrate a difference in the short-term outcomes between the two groups. Lastly, the long-term neuro-developmental outcome data were not available for our cohort even though majority of studies have not shown an increased rate of adverse major neuro-developmental outcomes such as cerebral palsy, cognitive and language deficits and behavioral problems in children born following the use of IVF or intracytoplasmic sperm injection. Minor deficits cannot be ruled out as in most studies the evaluation was performed during infancy [31].

Despite these limitations, we believe that the information from this study has clinical implications. Providing appropriate counseling that adverse outcomes is related to the GA at birth rather than ART itself may be reassuring to the couple who are already living with the financial and psychological stress of using ART for conception. Multi-fetal pregnancies in general are associated with adverse perinatal outcomes whether conceived by ART or SC. In order to reduce the risk of multi-fetal pregnancies, it is vital to counsel couples regarding the obstetrical, perinatal neonatal risk of such a pregnancy so that they can make an informed decision regarding number of embryos that should be transferred per cycle. The success of ART intervention should be quantified in terms of maternal and neonatal outcomes rather than the number of embryos who survived the procedure.

## Conclusion

In our population of multiples ≤ 32 weeks GA, the mode of conception did not influence the rate of mortality or short-term neonatal morbidities. Further, research on long-term neuro-developmental outcomes of these infants is needed.

## Competing interests

The authors declare that they have no competing interests.

## Authors' contributions

VS designed the study, participated in the checking, analyzing and interpretation of the data, and drafting of the manuscript. Both HA and KS participated in the study design, collected data and had input in drafting of the manuscript. WY performed statistical analysis of the data and assisted in interpretation of the data and PS contributed to the study design, providing data from the local database and had input in drafting of the manuscript. All authors have read and approved the final manuscript.

## Pre-publication history

The pre-publication history for this paper can be accessed here:

http://www.biomedcentral.com/1471-2431/11/54/prepub
